# Treatment of Polymicrobial Osteomyelitis with Ceftolozane-Tazobactam: Case Report and Sensitivity Testing of Isolates

**DOI:** 10.1155/2016/1628932

**Published:** 2016-06-29

**Authors:** Jeffrey C. Jolliff, Jackie Ho, Jeremiah Joson, Arash Heidari, Royce Johnson

**Affiliations:** ^1^Kern Medical, 1700 Mount Vernon Avenue, Bakersfield, CA 93306-4018, USA; ^2^University of the Pacific School of Pharmacy & Health Sciences, 3601 Pacific Avenue, Stockton, CA 95211-0109, USA

## Abstract

*Stenotrophomonas maltophilia* is an inherently multidrug resistant (MDR) opportunistic pathogen with many mechanisms of resistance. SENTRY studies reveal decreasing sensitivities of* S. maltophilia* to trimethoprim-sulfamethoxazole and fluoroquinolones. Ceftolozane-tazobactam (Zerbaxa, Merck & Co., Inc.) a novel intravenous combination agent of a third-generation cephalosporin and *β*-lactamase inhibitor was demonstrated to have* in vitro *activity against many Gram-positive, Gram-negative, and MDR organisms. Data for ceftolozane-tazobactam's use outside of Food and Drug Administration (FDA) approved indications has been limited thus far to two case reports which demonstrated its efficacy in pan-resistant* Pseudomonas aeruginosa* pneumonia. Herein, we describe the first published case of treatment of MDR* S. maltophilia *in polymicrobial osteomyelitis with long-term (>14 days) ceftolozane-tazobactam and metronidazole. Ceftolozane-tazobactam may offer a possible alternative for clinicians faced with limited options in the treatment of resistant pathogens including MDR* S. maltophilia.*

## 1. Introduction

*Stenotrophomonas maltophilia* is an inherently multidrug resistant (MDR) opportunistic pathogen with many mechanisms of resistance which may challenge clinicians to find safe and effective treatment regimens. Antimicrobial resistance mechanisms include *β*-lactamase production, the presence of class 1 integrons and ISCR elements (resistance to trimethoprim-sulfamethoxazole, TMP-SMX), expression of quinolone resistance (*Qnr*) genes, and multidrug efflux pumps [1].

Although TMP-SMX is often regarded as the drug of choice with fluoroquinolones (FQs) as reasonable alternatives, SENTRY studies reveal decreasing sensitivities of* S. maltophilia* to TMP-SMX (96.0% to 94.5%) and levofloxacin (83.4% to 77.3%) [2–4]. Other options with historically good susceptibility profiles but rising resistance rates include ceftazidime, ticarcillin-clavulanate, and tetracyclines [1]. Therefore, knowledge of the activity of other compounds, including new agents, which might be effective in treating* S. maltophilia* is desirable.

**Figure 1:**
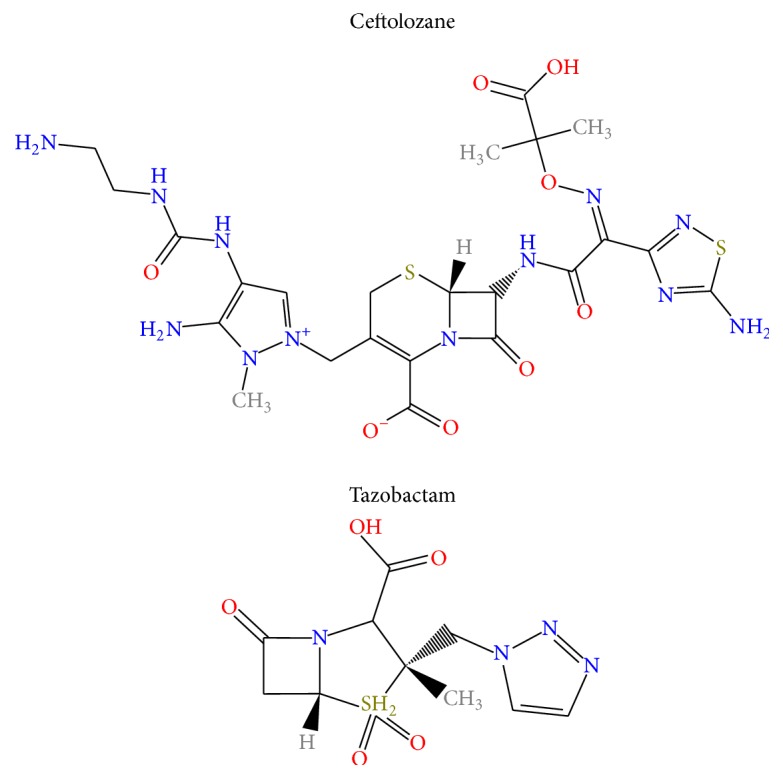
Chemical structure of ceftolozane-tazobactam.

Ceftolozane-tazobactam (Zerbaxa, Merck & Co., Inc.) is a novel intravenous combination agent of a third-generation cephalosporin and *β*-lactamase inhibitor Food and Drug Administration (FDA) approved in 2014 for the treatment of complicated intra-abdominal (cIAI) (when combined with metronidazole) and complicated urinary tract infection (cUTI) (Figure 1) [5]. Ceftolozane-tazobactam has demonstrated* in vitro *activity against many Gram-positive, Gram-negative, and MDR organisms. It retains activity against ESBL-producing Enterobacteriaceae (TEM-SHV, CTX-M, and OXA) and MDR* Pseudomonas aeruginosa* with resistance mechanisms including chromosomal AmpC, loss of outer membrane porin (OprD), and upregulation of efflux pumps (MexY and MexAB). Its activity against MDR* P. aeruginosa *is surpassed only by colistin [6].

Data for ceftolozane-tazobactam's use outside of the FDA approved indications (cIAI and cUTI) has been limited thus far to two case reports which demonstrated its efficacy in pan-resistant* P. aeruginosa* pneumonia [7, 8].

Herein, we describe the first published case of treatment of MDR* S. maltophilia *in polymicrobial osteomyelitis with long-term (>14 days) ceftolozane-tazobactam and metronidazole.

## 2. Case Presentation

A 20-year-old male with no significant past medical history presented to the emergency department after suffering a crush injury to his right foot. After incision and drainage (I&D) of the wound and open reduction internal fixation of the navicular, tarsal, and metatarsals, he was discharged on cephalexin 500 mg orally (PO) every 6 hours.

Subsequent clinic visits revealed delayed wound healing and moderate-to-severe edema. By postoperative week six, the wound had dehisced with signs of necrosis and abscess formation. He underwent surgical intervention the following day where the wound was incised and drained, hardware was removed, and cultures were obtained. He was sent home on levofloxacin 750 mg PO daily and told to follow up in one week. Upon return, inspection of his foot showed exposed metatarsal bone which was dark and foul smelling. He was admitted for further management.

Wound cultures taken at surgery the week prior resulted in* Klebsiella pneumoniae*,* Enterobacter cloacae*,* Streptococcus anginosus*, and* Bacteroides ovatus*. Piperacillin-tazobactam 3.375 g IV every 6 hours was started. Another I&D was performed with cultures taken from necrotic bone. A PICC line was placed to initiate a prolonged course of antimicrobials.

On day 4, bone cultures returned* S. anginosus*,* Granulicatella adiacens*, and MDR* S. maltophilia*, resistant to TMP-SMX and levofloxacin. Etests were ordered to explore alternative antimicrobial options: ceftolozane-tazobactam, minimum inhibitory concentration (MIC) 0.5 mg/L; tigecycline MIC 2 mg/L; ceftazidime MIC 2 mg/L; and colistin MIC 0.5 mg/L. Due to concerns that monotherapy would not suffice and the need for simplified outpatient parenteral antimicrobial therapy to facilitate patient discharge, ceftolozane-tazobactam was favored [9–11].

The antibiotic regimen was therefore changed to ceftolozane-tazobactam 1.5 g IV every 8 hours plus metronidazole 500 mg PO every 8 hours (for coverage against* Bacteroides ovatus*) for six weeks with wound VAC to be changed every other day. At six- and ten-week follow-up, his wound was noted to be healing nicely with no purulence or serous drainage; inflammatory symptoms were also absent. At week 14, granulation tissue had failed to completely cover exposed bone, so patient underwent reconstruction of right foot defect with a fasciocutaneous flap. Cultures were obtained from the excised subcutaneous tissue which resulted in pan-sensitive coagulase positive* Staphylococcus*,* Staphylococcus lugdunensis, *and* Gemella morbillorum*; however, antimicrobial therapy was forgone as no overt signs of infection were present. Also of note, this culture was negative for the previously cultured organisms (*S. anginosus*,* Granulicatella adiacens*, and* S. maltophilia*). At 30-week follow-up, fasciocutaneous graft had taken well and no signs of infection were present.

## 3. Discussion

Ceftolozane-tazobactam's many unique properties including the presence of a 7-aminothiadiazole (activity against Gram-negative organisms), alkoximino group (stability against *β*-lactamases), dimethylacetic acid moiety (activity against* P. aeruginosa*), and a bulky pyrazole ring (stability in the presence of AmpC *β*-lactamase) allow for increased activity against broad-spectrum Gram-negative organisms including some ESBL-producing Enterobacteriaceae and MDR* P. aeruginosa* [5, 6].

Ceftolozane-tazobactam's activity against these MDR Gram-negative pathogens in cIAI and cUTI is promising to clinicians. However, further information regarding its utility for off-label indications is speculative at best and based mostly upon unpublished manufacturer data from Phase I and II trials. In the case of osteomyelitis, variable bone to plasma ratio has been seen in animal data ranging from 5.2% to 9.0% in rabbit model and up to 40.0% in rat model femur concentration (Table 1). Nonetheless these ranges of results are comparable to cephalosporins that are widely used in osteomyelitis such as cefazolin and cefepime which have bone concentrations at 17.9% and 46%–76%, respectively (Table 2) [12]. While there are no Clinical & Laboratory Standards Institute (CLSI) approved MIC to predict sensitivity to* S. maltophilia, *one may speculate that a breakpoint of 0.5 mg/L may offer a reasonable chance of treatment success.

In this patient case, ceftolozane-tazobactam was demonstrated* in vitro* to be active against the offending pathogens including MDR* S. maltophilia. *The wound evidenced healing during and after completing antibiotic therapy and at posttreatment follow-up visits. Tolerability to ceftolozane-tazobactam beyond 14 days of treatment has previously not been demonstrated but was well tolerated in this case with no adverse drug reactions.

While further investigations are needed to examine ceftolozane-tazobactam's utility in off-label indication, the authors felt that it was important to share this experience with ceftolozane-tazobactam in this case of polymicrobial osteomyelitis. Ceftolozane-tazobactam may offer a possible alternative for clinicians faced with limited options in the treatment of resistant pathogens including MDR* S. maltophilia.*

## Figures and Tables

**Table 1:** Ratio of ceftolozane concentrations between bone tissue and plasma.

	Dose	Time after last dose (hours)	Bone : serum concentration ratio
Rabbit model	1 g q 8 h	1.5	*Marrow*: 14.1%–17.5% *Bone*: 6.2%–9.0%
Rat model	20 mg/kg	2	27%
		8	40%

*Source*: unpublished manufacturer data.

**Table 2:** Bone penetration of cephalosporins and *β*-lactamase inhibitors [12].

	Time after last dose (hours)	Bone (mg/kg): serum concentration (mg/L) ratio^a^	Method
Cephalosporins			
Ceftriaxone	0.2–8	0.07–0.17	HPLC
Cefotaxime	0.75–4	0.02–0.28	Bioassay
Cefuroxime (osteomyelitis)	1	0.04–0.08	HPLC
Cefazolin	0.9	0.179	HPLC
Cefepime	1-2	0.46–0.76	HPLC
Ceftazidime (ischemic bone)	1-2	0.04–0.08	HPLC
Ceftazidime	2	0.54	Bioassay

*β*-Lactamase inhibitors			
Clavulanic acid	1	1.14–1.76	Bioassay
Sulbactam	0.25–4	0.17–0.71	Gas chromatography
*Tazobactam*	*1.5*	*0.22–0.26*	*HPLC*

^a^Assumed bone density of 1 kg/L was assumed if not reported.

HPLC: high-performance liquid chromatography.

## References

[1] Chang Y.-T., Lin C.-Y., Chen Y.-H., Hsueh P.-R. (2015). Update on infections caused by Stenotrophomonas maltophilia with particular attention to resistance mechanisms and therapeutic options. *Frontiers in Microbiology*.

[2] Falagas M. E., Valkimadi P.-E., Huang Y.-T., Matthaiou D. K., Hsueh P.-R. (2008). Therapeutic options for *Stenotrophomonas maltophilia* infections beyond co-trimoxazole: a systematic review. *Journal of Antimicrobial Chemotherapy*.

[3] Farrell D. J., Sader H. S., Jones R. N. (2010). Antimicrobial susceptibilities of a worldwide collection of *Stenotrophomonas maltophilia* isolates tested against tigecycline and agents commonly used for *S. maltophilia* infections. *Antimicrobial Agents and Chemotherapy*.

[4] Sader H. S., Flamm R. K., Jones R. N. (2013). Tigecycline activity tested against antimicrobial resistant surveillance subsets of clinical bacteria collected worldwide (2011). *Diagnostic Microbiology and Infectious Disease*.

[5] Cho J. C., Fiorenza M. A., Estrada S. J. (2015). Ceftolozane/tazobactam: a novel cephalosporin/*β*-lactamase inhibitor combination. *Pharmacotherapy*.

[6] Cluck D., Lewis P., Stayer B., Spivey J., Moorman J. (2015). Ceftolozane-tazobactam: a new-generation cephalosporin. *American Journal of Health-System Pharmacy*.

[7] Soliman R., Lynch S., Meader E. (2015). Successful ceftolozane/tazobactam treatment of chronic pulmonary infection with pan-resistant *Pseudomonas aeruginosa*. *JMM Case Reports*.

[8] Alqaid A., Dougherty C. K., Ahmad S. (2015). Triple antibiotic therapy with ceftolozane/tazobactam, colistin and rifampin for pan-resistant Pseudomonas aeruginosa ventilator-associated pneumonia. *SWRCCC*.

[9] Garcia Sanchez J. E., Vazquez Lopez M. L., Blazquez De Castro A. M. (1997). Aztreonam/clavulanic acid in the treatment of serious infections caused by *Stenotrophomonas maltophilia* in neutropenic patients: case reports. *Journal of Chemotherapy*.

[10] Leung C., Drew P., Azzopardi E. A. (2010). Extended multidrug-resistant *Stenotrophomonas maltophilia* septicemia in a severely burnt patient. *Journal of Burn Care and Research*.

[11] Pérez P. N., Ramírez M. A., Fernández J. A., de Guevara L. L. (2014). A patient presenting with cholangitis due to *Stenotrophomonas maltophilia* and *Pseudomonas aeruginosa* successfully treated with intrabiliary colistine. *Infectious Disease Reports*.

[12] Landersdorfer C. B., Bulitta J. B., Kinzig M., Holzgrabe U., Sörgel F. (2009). Penetration of antibacterials into bone: pharmacokinetic, pharmacodynamic and bioanalytical considerations. *Clinical Pharmacokinetics*.

